# Reports of Baetidae (Ephemeroptera) species from Tafna Basin, Algeria and biogeographic affinities revealed by DNA barcoding

**DOI:** 10.3897/BDJ.8.e55596

**Published:** 2020-08-14

**Authors:** Nadhira Benhadji, Michel Sartori, Karima Abdellaoui Hassaine, Jean-Luc Gattolliat

**Affiliations:** 1 Laboratoire de recherche Valorisation des actions de l'homme pour la protection de l'environnement et application en santé publique, Université de Tlemcen, BP 119 13000, Tlemcen, Algeria Laboratoire de recherche Valorisation des actions de l'homme pour la protection de l'environnement et application en santé publique, Université de Tlemcen, BP 119 13000 Tlemcen Algeria; 2 Département d'Ecologie et Evolution, Université de Lausanne, Lausanne, Switzerland Département d'Ecologie et Evolution, Université de Lausanne Lausanne Switzerland; 3 Musée cantonal de zoologie, Lausanne, Switzerland Musée cantonal de zoologie Lausanne Switzerland

**Keywords:** Mayflies, *
Baetis
*, *
Rhodobaetis
*, *
Cloeon
*, DNA Barcoding, COI, endemism, Algeria

## Abstract

**Background:**

The Mediterranean basin is known to be the cradle of many endemic species. Within mayflies (Insecta, Ephemeroptera), North African species belonging to the family Baetidae remain poorly known and, traditionally, affinities to European fauna were proposed. Recent studies, based on molecular reconstructions, showed closer relationships to Mediterranean islands fauna.

**New information:**

Baetidae were sampled from North-West Algerian wadis (Tafna basin) and involved in COI barcoding reconstructions. Seven species were identified. The subgenus Rhodobaetis is represented by *Baetis atlanticus* known previously from Macaronesian islands, Europe and Morocco and the Maghrebian endemic *Baetis sinespinosus*. Specimens, previously identified as Cloeon cf. dipterum, correspond to *Cloeon peregrinator* and, until now, only reported from Macaronesia. Besides the confirmation of endemicity of some species, such as *Procloen stagnicola* and *B. sinespinosus*, our molecular study showed quite original results for relationships between European, insular and Algerian species. *Baetis maurus* stood out as a North African endemic sister clade to an Iberian clade. Furthermore, we found clear interspecific distances between Algerian and European clades for A. cf. sinaica and B. cf. pavidus, suggesting the presence of cryptic species in Algeria. However, additional studies are needed, as, for the moment, no clear morphological characters were found to separate the different clades and support them as valid species.

## Introduction

The family Baetidae has a cosmopolitan distribution and represents a quarter of the Ephemeroptera diversity worldwide both at generic and specific levels (Barber-James et al. 2008, Gattolliat and Nieto 2009, Sartori and Brittain 2015). The genera *Baetis* Leach, 1815 and *Cloeon* Leach, 1815 have the largest distribution amongst the family and encompass, respectively, 152 and 74 species (Sartori and Brittain 2015, Jacobus et al. 2019). In Europe, *Baetis* was originally divided into eleven species groups (Müller-Liebenau 1969), of which some are now considered as valid genera (*Alainites* Waltz and McCafferty, 1994; *Labiobaetis* Novikova and Kluge, 1987; *Nigrobaetis* Novikova and Kluge, 1987) or subgenera (*Patites* Thomas and Dia, 1999; *Rhodobaetis* Jacob, 2003) (Jacob 2003, Novikova and Kluge 1987, Thomas and Dia 2000, Waltz et al. 1994).

The subgenus Rhodobaetis (corresponding to the *Baetis rhodani* group) presently encompasses 43 species, some of them being amongst the most common and abundant mayflies. While some species are widely distributed (e.g. *Baetis rhodani* (Pictet, 1843), *Baetis atlanticus* Soldán and Godunko, 2006), others present a presumably restricted distribution, such as endemic to a single Canary Island (*Baetis palmensis* Gattolliat and Sartori, 2018; *B. tenerifensis* Gattolliat and Sartori, 2018; *B. gomerensis* Gattolliat and Sartori, 2018) or known from a restricted area (*Baetis chelif* Soldán, Godunko and Thomas, 2005 or *Baetis sinespinosus* Soldán and Thomas, 1983 in Algeria) (Gattolliat et al. 2018b, Soldán et al. 2005). Previous molecular studies, based on COI, revealed a high number of independent lineages, which may correspond to cryptic undescribed species (Bisconti et al. 2018, Bisconti et al. 2016, Gattolliat et al. 2015, Gattolliat et al. 2018b, Sroka 2012, Williams et al. 2006).

The species delimitation within the genus *Cloeon* Leach, 1815 (sensu Kluge 2016b) is also highly problematic. Most of the reports of the widely-distributed *Cloeon dipterum* (Linnaeus, 1761) or *Cloeon cognatum* Stephens, 1835 must be considered with caution as several independent lineages are hidden behind these concepts (Rutschmann et al. 2014, Rutschmann et al. 2017).

Thomas (1998) proposed the first preliminary checklist of mayflies from North Africa, including 25 Baetidae species. Eleven species are endemic to North Africa: *Centroptilum algericum* Eaton, 1899; *Baetis sinespinosus*; *Cloeon saharense* Soldán and Thomas, 1983; *Nigrobaetis numidicus* (Soldán and Thomas, 1983); *Nigrobaetis rhithralis* (Soldán and Thomas, 1983); *Procloeon stagnicola* Soldán and Thomas, 1983; *Cheleocloeon dimorphicum* (Soldán and Thomas, 1985); *Baetis berberus* Thomas, 1986; *Alainites oukaimeden* (Thomas and Sartori, 1992); *Alainites sadati* Thomas, 1994 and *Baetis chelif*. Eleven species are originally described from Algeria, ten of them between 1983 and 1986 (Soldán and Thomas 1983a, Soldán and Thomas 1983b, Soldán and Thomas 1985) and no new taxa have been described since 2005 (Soldán et al. 2005). Ten Central European species were reported from Maghreb, but all these identifications should be taken with caution. Remaining species present West Mediterranean distribution including North Africa and Iberian Peninsula (*Baetis punicus* Thomas, Boumaiza and Soldán, 1983; *Baetis maurus* Kimmins, 1938) or Italian peninsula and South of France (*Baetis pavidus* Grandi, 1949).

Recently, molecular reconstructions involving Baetidae were conducted for different projects, in particular for the origin of Macaronesian and Corsican mayflies fauna (Gattolliat et al. 2015, Gattolliat et al. 2018b, Rutschmann et al. 2014, Rutschmann et al. 2017). Despite being not directly focused on North Africa, they included specimens from Tunisia, Algeria and Morocco. These preliminary results for North Africa underlined important links between North African and Macaronesian faunas (in the case of *Cloeon* and *Baetis*). The discovery in Tunisia of a species of Leptophlebiidae, assumed as endemic to Sardinia (Zrelli et al. 2011), also confirmed possible connections between Italy and Maghreb, mainly during crucial geological events, such as the Messinian Salinity Crisis (Gattolliat et al. 2015).

North African species of *Labiobaetis* and *Cheleocloeon* Wuillot and Gillies, 1993 have most probably an Afrotropical origin as they are mainly diversified in this area. A dozenspecies of *Cheleocloeon* are described in Afrotropics, while the genus is only represented in the Palearctic by a single Maghrebian species (*Cheleocloeon dimorphicum*) and one in the Arabian Peninsula, *Cheleocloeon soldani* Gattolliat and Sartori, 2008 (Gattolliat and Sartori 2008, Kluge 2016a). Despite also being present in Central Europe, *Labiobaetis* is mostly diversified in tropical areas, as proven by its high diversity in Afrotropics (Gattolliat 2001, Lugo-Ortiz and McCafferty 1997) and South East Asia (Kaltenbach and Gattolliat 2018, Kaltenbach and Gattolliat 2019, Kaltenbach and Gattolliat 2020). While a part of the Afrotropical species present a very restricted distribution (Gattolliat 2001, Lugo-Ortiz and McCafferty 1997), a recent molecular reconstruction proved that specimens from Comoro Islands, South Africa and Arabian Peninsula form a monophyletic clade corresponding to *Labiobaetis glaucus* (Agnew, 1961) (Gattolliat et al. 2018a).

The present study is the first molecular analysis for Algerian mayflies using the cytochrome oxidase subunit I (COI) region for species delimitation. The main aims are to clarify the status of the different species of Baetidae present in North West Algeria, especially for Central European species assumed to occur in Maghreb. We also want to clarify the species delimitation in some groups with potential cryptic species and significantdifficulties to identify, based on morphological characters only. Finally, we want to understand the affinities between Maghrebian and neighbouring fauna.

## Materials and methods

### Sampling

**Figure 1.**
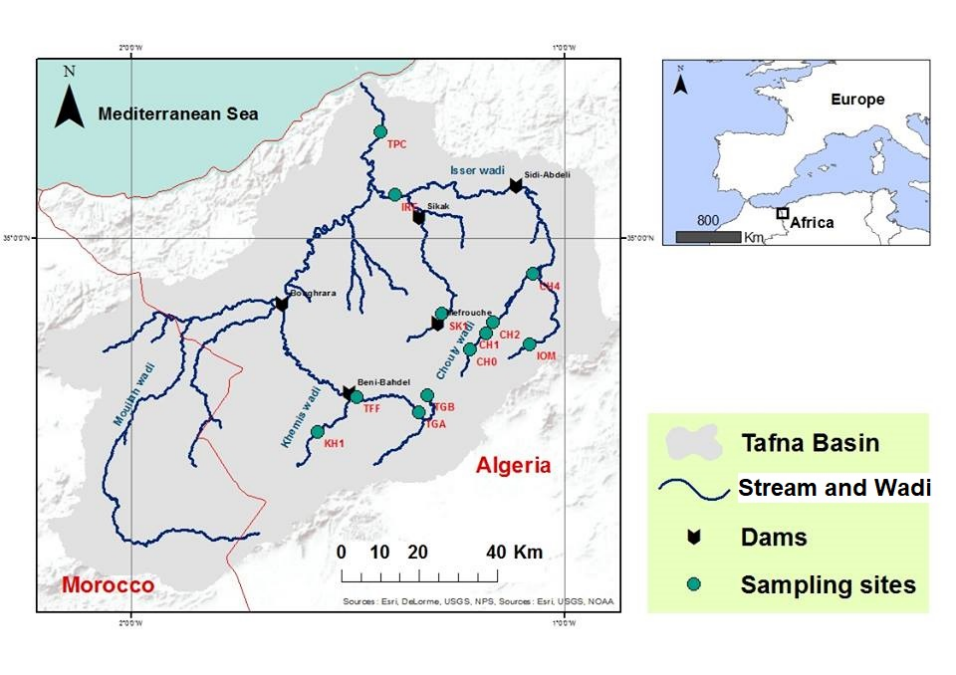
Location of the sampling sites (Tafna basin, Algeria).

We investigated twelve sampling sites, all located in the Tafna basin in North-West Algeria (Fig. 1); a detailed description of this area is presented in Benhadji et al. (2019). The Baetidae specimens used for the molecular study are listed in Table 1. They were collected by using a Surber net between April and October 2016, then preserved in 99% ethanol and stored at cold and stable temperature (4°C). A total of 52 larvae were identified at the generic or specific level, based on morphological characters (Benhadji et al. 2019), including *Rhodobaetis* spp. (26 specimens), B. cf. pavidus (13 specimens), *B. maurus* (four specimens), Acentrella cf. sinaica (one specimen), C. cf. dipterum (four specimens) and *P. stagnicola* (three specimens) (Table 1). Specimens and DNA extractions are housed in the collections of the Museum of Zoology, Lausanne, Switzerland.

### COI gene amplifications

We performed DNA extraction using DNeasy Blood & Tissue kit (QIAGEN) and BioSprint 96 extraction robot (Qiagen) by soaking each specimen in buffer and proteinase K at 56°C for an overnight incubation. The mitochondrial DNA cytochrome oxidase c subunit I gene (COI) was amplified using the primers LCO1490 and HCO2198 (Folmer et al. 1994) with an initial denaturation temperature of 98°C for 30 sec followed by a total of 37 cycles with denaturation temperature of 98°C for 10 sec, an annealing temperature of 50°C for 30 sec and an extension at 72°C for 30 sec, final extension at 72°C for 2 min. We checked if the amplification was successful using agarose gel electrophoresis, then we purified PCR products and prepared bi-directional sequencing using the same primers LCO1490 and HCO2198.

### COI gene trees

We corrected and edited forward and reverse sequencing reads using Bioedit, then we assembled each of the two complementary sequences using Codon Code Aligner (demo mode) and obtained sequence alignments (Suppl. materials 4, 5, 6, 7, 8, 9) using Jalview 2.10.1 via Mafft alignment as in Vuataz et al. (2011). We aligned sequences of each taxon with analogue genus or species selected from Genbank database (GenBank 2019) or BoldSystem database (BOLD 2019). For *Rhodobaetis*, we added sequences corresponding to the known haplogroups of *Baetis rhodani* (Gattolliat et al. 2015, Gattolliat et al. 2018b, Lucentini et al. 2011, Williams et al. 2006). For *B. maurus*, we selected all available sequences of *B. maurus* and several of B. gr. alpinus. For the remaining taxa, in addition to conspecific sequences, we incorporated outgroups sequences (for example, *Cloeon simile* Eaton, 1870, *C. praetextum* Bengtsson, 1914 and *C. smaeleni* Lestage, 1924 to C. cf. dipterum reconstruction). To delimit the haplogroups, we used ABGD, Automatic Barcode Gap Discovery (Puillandre et al. 2012).

To reconstruct the trees, we used Mega version 10.0.4; we chose the best evolutionary model using the AICc criteria (Akaike 1974), then we set to run a Maximum Likelihood bootstrap analysis with 1000 normal bootstrap replicates.

## Checklists

### Checklist of the Baetidae species of the Tafna basin (North-West Algeria)

#### Ephemeroptera

Hyatt & Arms, 1890

015A1E71-E333-5221-9814-225FF797BA68

#### Baetidae

Leach, 1815

3FAF161E-2364-543C-B25B-13A942C6785C

#### Acentrella

Bengtsson, 1912

70EF0C3E-5A11-5940-9607-A74E9CC6683F

#### Acentrella cf. sinaica

Bogoescu, 1931

EAE1FE9C-ECED-5F31-88AE-F82D05D2F53D

#### Baetis

Leach, 1815

ACF7B2E0-1AD0-594A-969D-6DCE74853EB1

#### Baetis (Rhodobaetis) atlanticus

Soldán and Godunko 2006

178EF1C3-F5F6-5242-ABFA-9C3920A9B996

#### Baetis maurus

Kimmins, 1938

C7233785-EEA5-589D-845D-7CCB25F3FA60

#### Baetis cf. pavidus

Grandi, 1949

AB6B940D-255C-5B41-AC6D-B030AD61599E

#### Baetis (Rhodobaetis) sinespinosus

Soldán and Thomas, 1983

89613628-8222-5FEB-8ACD-97F834398E20

#### Cloeon

Leach, 1815

FA06CCC9-0475-5137-9079-C70775C6EF19

#### Cloeon peregrinator

Gattolliat and Sartori, 2008

CA10A785-8255-5D83-A0AE-88209FE89D12

#### Procloeon

Bengtsson, 1915

CB811D74-9C3B-56F9-9DED-8398387C7667

#### Procloeon stagnicola

Soldán and Thomas, 1984

40E16844-D041-5061-B5B0-2083F07193EF

## Analysis

**Species delimitations**

Based on the molecular analysis, seven Baetidae species were recognised or confirmed in the Tafna basin sites.

**Subgenus Rhodobaetis**

**Figure 2.**
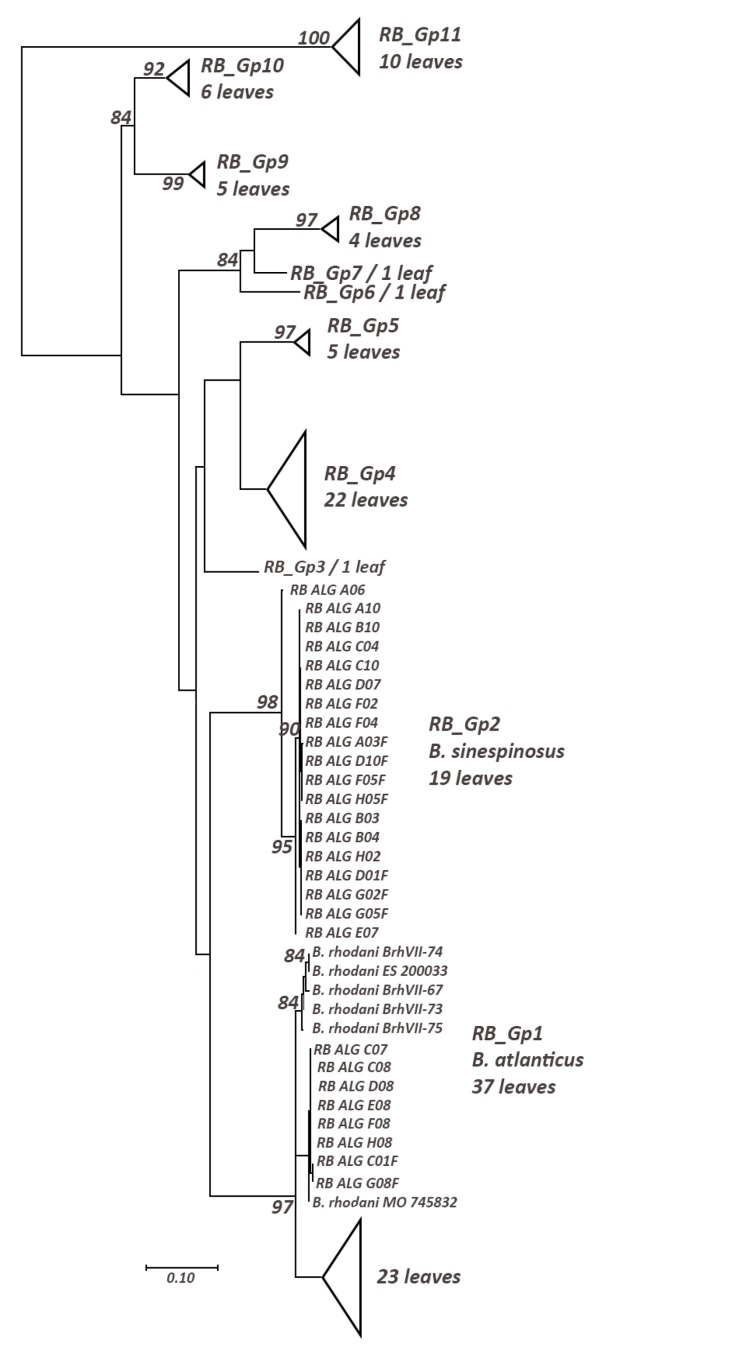
Maximum Likelihood tree including representative of *Rhodobaetis* using TN93 (+G+I) model; only bootstrap supports (BS) higher than 70% are indicated on the corresponding branch.

We obtained 11 haplogroups of *Rhodobaetis* (Suppl. material 1, Table 2) with the 27 Algerian sequences differentiating into two haplogroups (Fig. 2): a) RB_Gp1, a strongly supported monophyletic clade (97% BS) also containing Macaronesian and Iberian sequences from *B. atlanticus*; b) RB_Gp2, a monophyletic clade with a strong bootstrap support (98%) containing only Algerian sequences of *B. sinespinosus*. RB_Gp1 and RB_Gp2 have, respectively, 1.65% and 0.48% of intraspecific distance. RB_Gp1 and RB_Gp2 are poorly supported as a monophyletic clade (25% BS); the two haplogroups present an interspecific distance of 15.2%.

**Baetis cf. pavidus**

**Figure 3.**
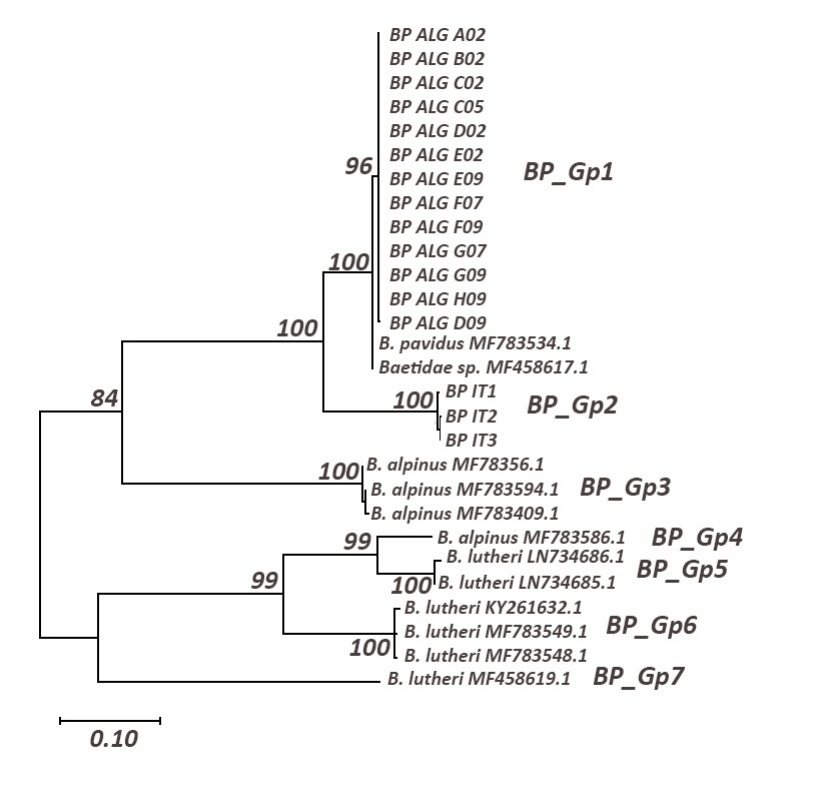
Maximum Likelihood tree including a representative of *Baetis pavidus* using the General Time Reversible model (+G+I); only bootstrap supports (BS) higher than 70% are indicated on the corresponding branch.

We obtained two haplogroups of Baetis cf. pavidus (Fig. 3, Table 3): a highly-supported clade gathering Algerian specimens and two sequences from southern France (BP_Gp1) and a second clade (BP_Gp2) which is composed by *Baetis pavidus* from Italy. Both haplogroups are highly supported as sister clades. BP_Gp1 possesses a very low intraspecific distance (0.2%) and high interspecific distance with its sister clade BP_Gp2 (11%).

***Baetis maurus***

**Figure 4.**
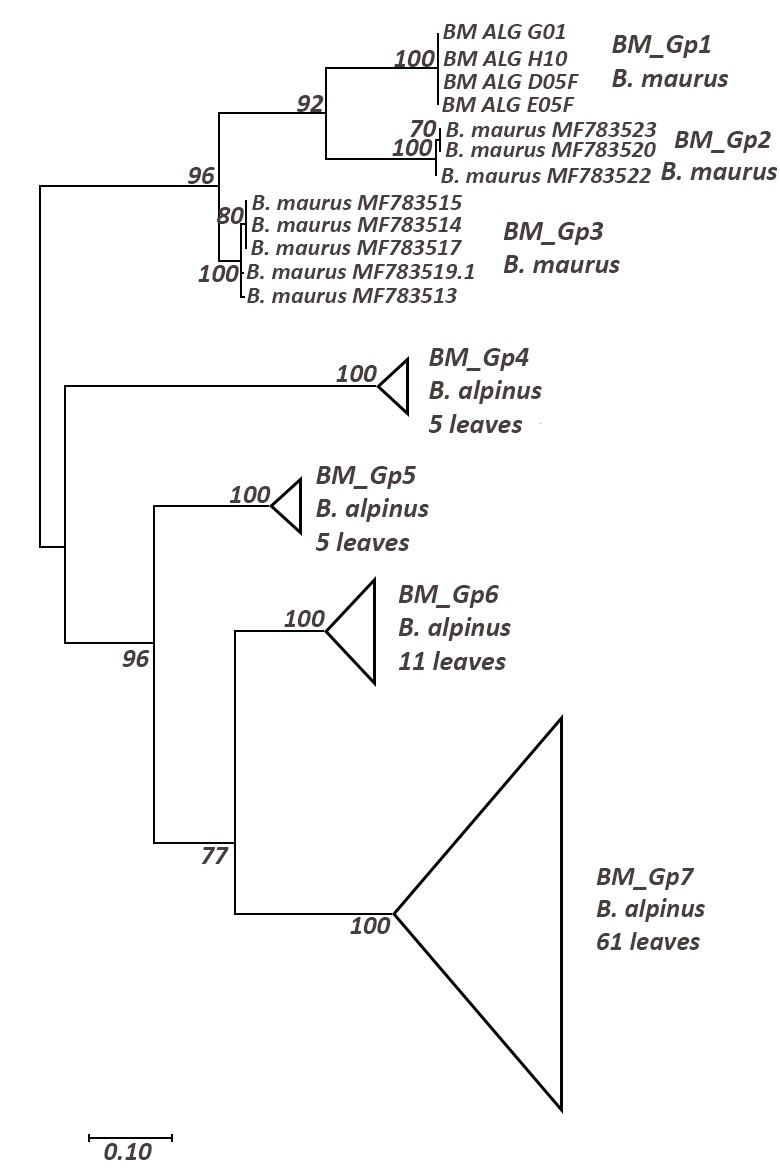
Maximum Likelihood tree including a representative of *Baetis maurus* using TN93 (+G+I) model; only bootstrap supports (BS) higher than 70% are indicated on the corresponding branch.

We delineated three *B. maurus* haplogroups (Suppl. material 2, Table 4). Our sequences (BM_Gp1) belong to a strongly-supported monophyletic haplogroup identified as *Baetis maurus* (Fig. 4). This haplogroup is highly supported as the sister clade of specimens identified also as *B. maurus* (BM_Gp2 and BM_Gp3), but coming from Spain; the distances between these clades, all identified as *B. maurus*, are of interspecific level (15-16%).

**Acentrella cf. sinaica**

**Figure 5.**
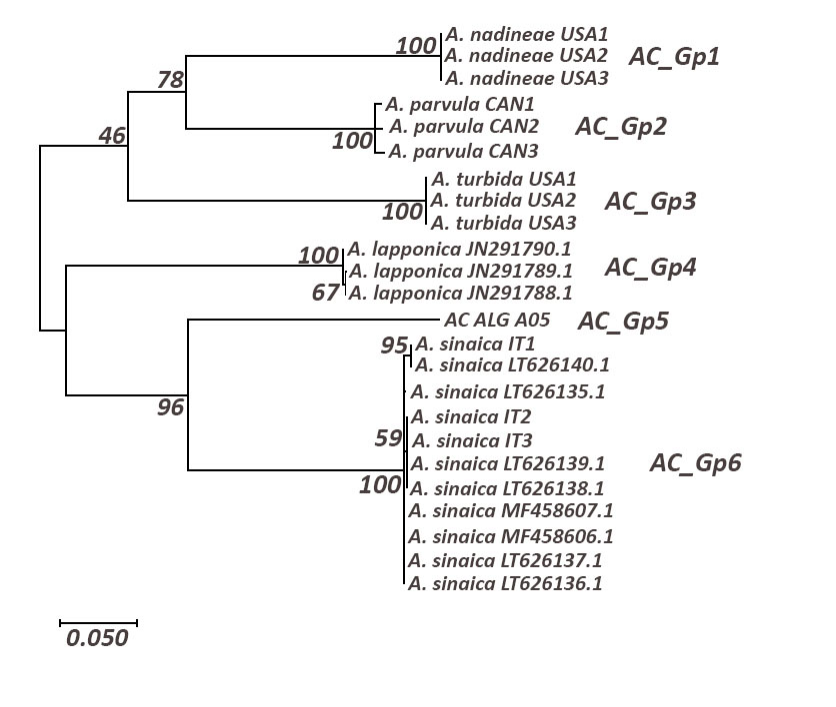
Maximum Likelihood tree including a representative of *Acentrella* spp using the General Time Reversible model (+I); only bootstrap supports (BS) higher than 70% are indicated on the corresponding branch.

The reconstruction divided *Acentrella* sequences into 6 haplogroups (Fig. 5, Table 5). The single sequence from Algeria was recovered as an independent clade (AC_Gp5) distant at least 19% from other clades. AC_Gp6 haplogroup is the sister clade of AC_Gp5; it contains *A. sinaica* sequences from France and Italy gathered with a very strong BS (100%).

***Cloeon peregrinator***

We obtained 11 haplogroups from the reconstruction (Suppl. material 3, Table 6) including six highly supported C. cf. dipterum haplogroups. The clade containing all haplogroups of *C. dipterum* s.l. is a monophyletic clade with a high BS (99%).

**Figure 6.**
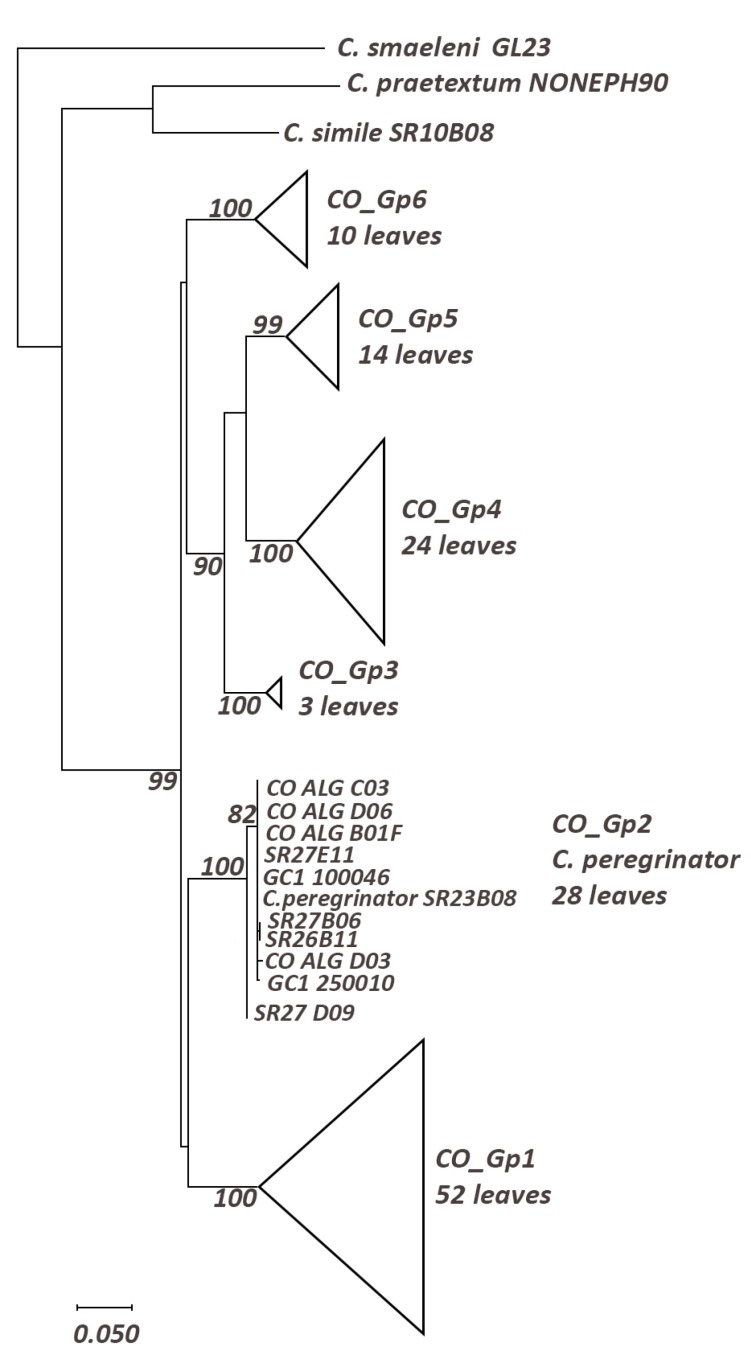
Maximum Likelihood tree including a representative of *Cloeon* spp. using the General Time Reversible model (+G+I); only bootstrap supports (BS) higher than 70% are indicated on the corresponding branch.

The CO_Gp2 haplogroup, which includes four sequences from Algeria and sequences of *C. pereginator* from Madeira (type locality) and Gran Canaria (Fig. 6), is highly supported as a monophyletic haplogroup (100%). This haplogroup has a low intraspecific distance (0.2%) and high interspecific distance with all the other haplogroups of *C. dipterum* s.l., for instance with CO_Gp1 with which it has the least distance (8.9%). Consequently, the sequences from Algeria and Gran Canaria belong to *C. peregrinator*.

***Procloeon stagnicola***

**Figure 7.**
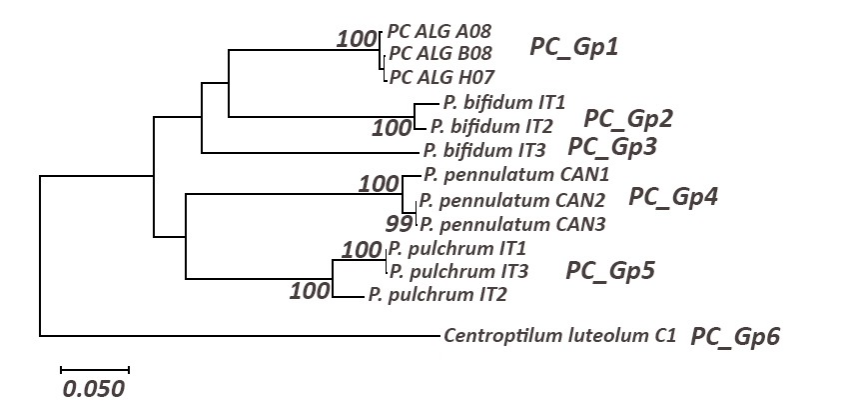
Maximum Likelihood tree including a representative of *Procloeon* spp using the General Time Reversible model (+G+I); only bootstrap supports (BS) higher than 70% are indicated on the corresponding branch.

In this reconstruction, six haplogroups (Fig. 7, Table 7) were obtained. *Procloeon stagnicola* from Algeria (PC_Gp1) forms a well-supported monophyletic haplogroup. PC_Gp1 has a high interspecific distance from PC_Gp2 (16.2%), which corresponds to the closest European species *Procloeon bifidum* (Bengsston, 1912) and from the remaining sequences (from 16% to 23.7%).

## Discussion

The different trees we obtained allowed us to better understand the composition of Algerian Baetidae. Based on our analysis, we can link the Algerian lineages with their sister-groups, calculate the maximum and minimum distances and evaluate which lineages may represent putative species.

**Maghrebian endemic species**

**Baetis (Rhodobaetis) sinespinosus Soldán and Thomas, 1983**

Rhodobaetis is a subgenus of Baetis and corresponds to the concept of *Baetis rhodani* species-group (Jacob 2003, Müller-Liebenau 1969). It is widely distributed in all West Palearctic streams (Gattolliat et al. 2015). Three species of *Rhodobaetis* are reported from Algeria, two of them are endemic: *Baetis chelif* and *Baetis sinespinosus* (Bauernfeind and Soldán 2012, Thomas 1998). A representative of *B. rhodani* s.l. is also reported (Thomas 1998). As noticed by Sroka (2012), it is generally difficult to find morphological characters to support the molecular species delimitation within *Rhodobaetis*. In the present case, the absence of a single rudimentary scale on the tip of the maxillary palp and the presence of four rows of setae at the apex of the paraglossae indicate that the clade RB_Gp2 corresponds to *B. sinespinosus.Baetis sinespinosus* is a well-supported monophyletic clade and presents high interspecific distances with all the other European and Mediterranean species. According to our data and reports from literature, this species seems to be endemic to Algeria. However, its presence in nearby countries, such as Tunisia and Morocco, will not be surprising as several populations were provisionally identified as *Baetis rhodani* s.l. (Mabrouki et al. 2017, Thomas 1998, Zrelli et al. 2016) and the present study is based on material collected very close to the border of Morocco.

***Procloeon stagnicola* Soldán and Thomas, 1983**

Our results showed a high interspecific distance between the Algerian clade and its European sister species *Procloeon bifidum*; thus, it confirms the validity of *Procloeon stagnicola*. This latter differs from P. bifidum especially by the flat and rounded bristles on the labrum margin; the pointed apex of the gills with an extremely reduced second lamella and also by the lateral margins of the abdominal segments which possess spines from segment V to IX. The species was originally described from Algeria (Soldán and Thomas 1983a) and was subsequently discovered in Tunisia (Boumaiza and Thomas 1995). The report of *Procloeon bifidum* from Morocco (El Alami et al. 2000) may be a misidentification and may also concern *P. stagnicola*. Identification of material collected by the last author (Jean-Luc Gattolliat) and stored in the MZL collection confirmed the presence of this species in Morocco (unpublished data). This species should be therefore considered as endemic to Maghreb.

***Baetis maurus* Kimmins, 1938**

*Baetis maurus* is a representative of the *Baetis alpinus* species-group. The species is considered as an Atlanto-Mediterranean element (Bauernfeind and Soldán 2012). It was originally described from Morocco (Kimmins 1938), then reported from the Iberian Peninsula (Alba-Tercedor 1982, Müller-Liebenau 1974). Algerian haplotypes present high distances with presumably conspecific specimens from Spain (Murria et al. 2017), as well as with the other Euro-Mediterranean species belonging to the *B. alpinus* species-group. Our results tend to prove that *B. maurus* is, in fact, a Magrebian endemic species (originally described from Morocco) and that at least one sister undescribed species occurs in the Iberian Peninsula. These preliminary results must be confirmed by sequencing additional populations from Spain and Maghreb and by morphological evidence. With the presence of a second rudimentary row of denticles on claws, both Maghrebian and Iberian populations possess a unique morphological character amongst the genus *Baetis* (Thomas et al. 1983).

**Acentrella cf. sinaica Bogoescu, 1931**

*Acentrella sinaica* is a South and Central European species (Bauernfeind and Soldán 2012). It was reported from Tunisia (Boumaiza and Thomas 1995) and then from Algeria (Mebarki et al. 2017). In his checklist of North African mayflies, Thomas (1998) considered the specific identification as possibly incorrect, referring maybe to *Acentrella almohades* Alba-Tercedor & El Alami 1999 described from Morocco (Alba-Tercedor and El Alami 1999). The important distances between our unique haplotype and those from France and Italy tend to confirm that Algerian specimens do not belong to *A. sinaica*. Unfortunately, no sequence is available for *A. almohades.* We refrain to attribute the specimens from Algeria to *A. almohades*, as important distinctive characters do not match between our specimens and the original description. Especially, the Algerian specimens possess long setae along the dorsal margin of the femora (similar to *A. sinaica*), while *A. almohades* present much shorter ones (Alba-Tercedor and El Alami 1999). There is, therefore, some probability that our specimens represent an undescribed species from North Africa. Additional sequences and close morphological studies are needed to confirm this hypothesis.

**Western Mediterranean species**

**Baetis cf. pavidus Grandi, 1949**

*Baetis pavidus* is a Western Mediterranean species and was originally described from Italy and then reported from the Maghreb (Bauernfeind and Soldán 2012, Jacob 2003). In Algeria and Tunisia, it is one of the most common and abundant species at low to middle elevation and is rather tolerant to pollution and low oxygenation (Benhadji et al. 2019, Boumaiza and Thomas 1995). The Algerian haplotypes present low genetic distance with specimens from southern France, confirming the link for this species between North African and South European populations. The sequences from Sicily, Italy (BP_Gp2) were not assigned to *Baetis pavidus* with certainty by Tenchini et al. (2018) and may represent an undescribed species close to *B. pavidus* or, alternatively, may be correctly associated with *B. pavidus* and the specimens from Algeria and from South of France represent a new species.

**Widely-distributed species**

***Cloeon peregrinator* Gattolliat and Sartori, 2008**

*Cloeon peregrinator* was first considered as an endemic species from Madeira (Gattolliat et al. 2008), then was also found on the Canary Islands (Rutschmann et al. 2017). It belongs to the *Cloeon dipterum* species-group from which it can be separated by minute morphological characters (Gattolliat et al. 2008). Algerian haplotypes present a low genetic distance with Madeiran specimens which prove their conspecificity. This discovery is rather surprising as *C. peregrinator* was first thought to be an insular endemic. Molecular studies showed mayflies are able to colonise islands, even for such taxa with presumably low dispersal capacity (Monaghan et al. 2005, Rutschmann et al. 2014). The origin of the species cannot yet be proven and two scenarios can be proposed: either a colonisation of Macaronesia from Continental Europe, then a speciation process on the islands and a subsequent colonisation of North Africa or, alternatively, a colonisation of North Africa by a European lineage followed by a speciation process and subsequently a colonisation of Macaronesia.

***Baetis atlanticus* Soldán and Godunko, 2006**

Bauernfeind and Soldán (2012) stated that *Baetis atlanticus* is a *Rhodobaetis* species endemic to Madeira, while Rutschmann et al. (2014) showed later that the species has a much wider distribution. They pointed out its European or North-African (Morocco) origin and suggested a recent colonisation of Madeira. *Baetis atlanticus* is widely distributed in Atlantic Europe as proven by the recent reports of the species from the United Kingdom (Macadam et al. 2018). This species remains difficult to distinguish morphologically from other *Rhodobaetis* species (Soldán and Godunko 2006). The main discriminating character between *B. atlanticus* and the North-African endemic *B. sinespinosus* is the number of regular rows of long setae at the apex of the paraglossae (the usual three rows in *B. atlanticus* and four rows in *B. sinespinosus*) and the apex of the maxillary palp (with one typical small apical scale in *B. atlanticus* and without the apical scale in *B. sinespinosus*). The genetic distances amongst Macaronesian, Iberian and Algerian specimens unequivocally confirm the conspecificity of the different populations. The ecological preference of *B. atlanticus* in the Tafna catchment is similar to that of the lowlands of Madeira (Soldán and Godunko 2006): larvae prefer coarse substrate composed of rocks, cobbles or pebbles in low, moderate to fast current velocities; they were less abundant and only present in relatively-preserved sites at higher altitude (CH0; CH1; KH1; SK1; IOM). In comparison, *B. sinespinosus* larvae have a much wider ecological range and are more pollution resistant; they are also highly abundant and present in all sampling sites with various substrates and velocities.

### Concluding remarks

We summarise the state of the knowledge and the implication of the present study for the Baetidae fauna of North-West Algeria in Table 8. As we mostly found interspecific distances between Algerian and European lineages, our results generally highlighted and confirmed the high endemism of North African Baetidae. According to the present knowledge, the endemism may be restricted to Algeria or to the Maghreb, even for species that were supposed to present West Palearctic distribution (Western Europe and North Africa). The link with the Iberian Peninsula is less strong than expected, as no species included in the study is shared only between the two areas. From a genetic point of view, only one case of sister-species was found (*Baetis maurus*). Baetis cf. pavidus is the only species with a West Mediterranean distribution, as the same species occurs in Algeria and South of France. Finally, our study confirms the presence of Macaronesian and Atlantic species in Maghreb and, therefore, endorses the preliminary results of Rutschmann et al. (2014), Rutschmann et al. (2017).

The next steps will be to sequence more specimens from different areas of Algeria and also from Morocco and Tunisia to confirm the monophyly of the different North African clades. The results, especially the validation of the new species hypotheses, need to be confirmed by integrative methods. Only morphological evidence and more mitochondrial or nuclear genes can validate the specific status of these clades. Our study may have implications outside of North Africa, as our results suggest that one or two lineages, previously supposed to belong to *Baetis maurus*, may represent new species in Spain, as well as the presumably non-conspecificity of the French and Italian lineages of *Baetis pavidus*.

## Supplementary Material

0AA9A55F-FA8D-5643-B15C-88D45585A472 10.3897/BDJ.8.e55596.suppl1 Supplementary material 1 Complete Maximum Likelihood tree including representative of *Rhodobaetis* using TN93 (+G+I) model Data type Biomolecular tree File: oo_420313.tif https://binary.pensoft.net/file/420313 Nadhira Benhadji, Michel Sartori, Karima Abdellaoui Hassaine & Jean-Luc Gattolliat

EA527427-F2CE-594A-A287-56B1257FA3BE 10.3897/BDJ.8.e55596.suppl2 Supplementary material 2 Complete Maximum Likelihood tree including representative of *Baetis maurus* using TN93 (+G+I) model Data type Biomolecular tree File: oo_420317.tif https://binary.pensoft.net/file/420317 Nadhira Benhadji, Michel Sartori, Karima Abdellaoui Hassaine & Jean-Luc Gattolliat

A5E013D0-5B1E-5DC2-AEA0-2EEE70CD5639 10.3897/BDJ.8.e55596.suppl3 Supplementary material 3 Complete Maximum Likelihood tree including representative of *Cloeon* spp. using General Time Reversible model (+G+I) Data type Biomolecular tree File: oo_420319.tif https://binary.pensoft.net/file/420319 Nadhira Benhadji, Michel Sartori, Karima Abdellaoui Hassaine & Jean-Luc Gattolliat

F062D8CC-D5F2-53AE-AC00-90C5F998A2A1 10.3897/BDJ.8.e55596.suppl4 Supplementary material 4 RB_Sequence alignment Data type Nucleotide sequences File: oo_435076.fasta https://binary.pensoft.net/file/435076 Nadhira Benhadji, Michel Sartori, Karima Abdellaoui Hassaine & Jean-Luc Gattolliat

1759F1E3-C62D-5312-9021-654B03A35765 10.3897/BDJ.8.e55596.suppl5 Supplementary material 5 BP_sequence alignment Data type Nucleotide sequence File: oo_435079.fas https://binary.pensoft.net/file/435079 Nadhira Benhadji, Michel Sartori, Karima Abdellaoui Hassaine & Jean-Luc Gattolliat

E598F730-1B85-5944-9100-4937C9CC46FD 10.3897/BDJ.8.e55596.suppl6 Supplementary material 6 BM_sequence alignment Data type Nucleotide sequences File: oo_435073.fas https://binary.pensoft.net/file/435073 Nadhira Benhadji, Michel Sartori, Karima Abdellaoui Hassaine & Jean-Luc Gattolliat

F9019652-05CB-5BE6-86B9-35B28FA54660 10.3897/BDJ.8.e55596.suppl7 Supplementary material 7 AC_sequence alignment Data type Nucleotide sequences File: oo_435075.fas https://binary.pensoft.net/file/435075 Nadhira Benhadji, Michel Sartori, Karima Abdellaoui Hassaine & Jean-Luc Gattolliat

4537A9A3-FE17-5167-9587-EF7B5C8A094F 10.3897/BDJ.8.e55596.suppl8 Supplementary material 8 CO_sequence alignment Data type Nucleotide sequences File: oo_435071.fas https://binary.pensoft.net/file/435071 Nadhira Benhadji, Michel Sartori, Karima Abdellaoui Hassaine & Jean-Luc Gattolliat

784D9D4C-109B-5E87-B042-C6BDF28BFD4E 10.3897/BDJ.8.e55596.suppl9 Supplementary material 9 PC_sequence alignment Data type Nucleotide sequences File: oo_435074.fas https://binary.pensoft.net/file/435074 Nadhira Benhadji, Michel Sartori, Karima Abdellaoui Hassaine & Jean-Luc Gattolliat

XML Treatment for Ephemeroptera

XML Treatment for Baetidae

XML Treatment for Acentrella

XML Treatment for Acentrella cf. sinaica

XML Treatment for Baetis

XML Treatment for Baetis (Rhodobaetis) atlanticus

XML Treatment for Baetis maurus

XML Treatment for Baetis cf. pavidus

XML Treatment for Baetis (Rhodobaetis) sinespinosus

XML Treatment for Cloeon

XML Treatment for Cloeon peregrinator

XML Treatment for Procloeon

XML Treatment for Procloeon stagnicola

## Figures and Tables

**Table 1.** List of COI sequenced specimens with Genbank accession number. For sites code, see Benhadji et al. (2019). Each specimen is identified by alphanumeric codes. The two first letters indicate the taxonomic group (RB: *Rhodobaetis*; BP: *Baetis pavidus*; BM: *Baetis maurus*; AC: Acentrella cf. sinaica; CO: *Cloeon peregrinator*; PC: *Procloeon stagnicola*). ALG indicates the country (i.e. Algeria). The following letter (A to H) with number (1 to 8) indicate the position of the well on the PCR plate. An “F” is added at the end of some codes when only the forward amplification was successful.

**Taxa**	**Sites**	**Codes**	**Genbank accession**
***Baetis sinespinosus***	SK1	RB_ALG_A06	MT800078
	SK1	RB_ALG_D07	MT800085
	SK1	RB_ALG_E07	MT800086
	SK1	RB_ALG_F05F	MT800093
	SK1	RB_ALG_G05F	MT800095
	SK1	RB_ALG_H05F	MT800096
	CH0	RB_ALG_D01F	MT800091
	CH1	RB_ALG_B03	MT800080
	CH1	RB_ALG_F02	MT800087
	CH1	RB_ALG_H02	MT800089
	CH1	RB_ALG_A03F	MT800090
	CH1	RB_ALG_G02F	MT800094
	CH4	RB_ALG_B04	MT800081
	CH4	RB_ALG_C04	MT800083
	IOM	RB_ALG_F04	MT800088
	TGB	RB_ALG_C10	MT800084
	TGB	RB_ALG_D10F	MT800092
	TFF	RB_ALG_A10	MT800079
	TFF	RB_ALG_B10	MT800082
***Baetis atlanticus***	SK1	RB_ALG_C07	MT800053
	CH0	RB_ALG_C01F	MT800059
	KH1	RB_ALG_C08	MT800054
	KH1	RB_ALG_D08	MT800055
	KH1	RB_ALG_E08	MT800056
	KH1	RB_ALG_H08	MT800058
	KH1	RB_ALG_F08	MT800057
	KH1	RB_ALG_G08F	MT800060
**Baetis cf. pavidus**	SK1	BP_ALG_F07	MT800069
	SK1	BP_ALG_G07	MT800071
	CH1	BP_ALG_B02	MT800062
	CH1	BP_ALG_C02	MT800063
	CH1	BP_ALG_D02	MT800065
	CH1	BP_ALG_E02	MT800067
	CH4	BP_ALG_A02	MT800061
	IOM	BP_ALG_C05	MT800064
	TGA	BP_ALG_D09	MT800066
	TGA	BP_ALG_E09	MT800068
	TFF	BP_ALG_F09	MT800070
	TFF	BP_ALG_G09	MT800072
	TFF	BP_ALG_H09	MT800073
***Baetis maurus***	CH1	BM_ALG_G01	MT800074
	CH1	BM_ALG_H10	MT800075
	SK1	BM_ALG_D05F	MT800076
	SK1	BM_ALG_E05F	MT800077
**Acentrella cf. sinaica**	IOM	AC-ALG-A05	MT800052
***Cloeon peregrinator***	CH0	CO_ALG_B01F	MT800100
	CH1	CO_ALG_C03	MT800097
	CH1	CO_ALG_D03	MT800098
	SK1	CO_ALG_D06	MT800099
***Procloeon stagnicola***	KH1	PC_ALG_A08	MT800101
	KH1	PC_ALG_B08	MT800102
	KH1	PC_ALG_H07	MT800103

**Table 2.** Distances within (in bold) and between *Rhodobaetis* haplogroups. RB-Gp1: Baetis (Rhodobaetis) atlanticus; RB-Gp2: Baetis (Rhodobaetis) sinespinosus. Haplogroups with Algerian sequences are underlined.

	**RB_Gp1**	**RB_Gp2**	**RB_Gp3**	**RB_Gp4**	**RB_Gp5**	**RB_Gp6**	**RB_Gp7**	**RB_Gp8**	**RB_Gp9**	**RB_Gp10**	**RB_Gp11**
**RB_Gp1**	**0.02**										
**RB_Gp2**	0.15	**0.004**									
**RB_Gp3**	0.16	0.14	**n/c**								
**RB_Gp4**	0.15	0.14	0.12	**0.004**							
**RB_Gp5**	0.15	0.14	0.14	0.10	**0.01**						
**RB_Gp6**	0.17	0.17	0.18	0.18	0.20	**n/c**					
**RB_Gp7**	0.18	0.16	0.17	0.14	0.16	0.12	**n/c**				
**RB_Gp_8**	0.20	0.19	0.17	0.17	0.20	0.14	0.12	**0.01**			
**RB_Gp_9**	0.18	0.16	0.15	0.17	0.20	0.19	0.18	0.21	**0.003**		
**RB_Gp10**	0.19	0.17	0.17	0.16	0.17	0.19	0.18	0.19	0.10	**0.01**	
**RB_Gp11**	0.26	0.24	0.23	0.23	0.25	0.22	0.23	0.25	0.23	0.22	**0.002**

**Table 3.** Distances within (in bold) and between Baetis cf. pavidus (BP_Gp1-BM_Gp2), *Baetis alpinus* (BP_Gp3) and *Baetis lutheri* haplogroups (BP_Gp4-BP_Gp7). Haplogroup with Algerian sequences is underlined.

	**BP_Gp1**	**BP_Gp2**	**BP_Gp3**	**BP_Gp4**	**BP_Gp5**	**BP_Gp6**	**BP_Gp7**
**BP_Gp1**	**0.002**						
**BP_Gp2**	0.11	**0.003**					
**BP_Gp3**	0.21	0.23	**0.004**				
**BP_Gp4**	0.22	0.24	0.23	**n/c**			
**BP_Gp5**	0.23	0.25	0.24	0.08	**0.006**		
**BP_Gp6**	0.23	0.25	0.25	0.15	0.15	**0.008**	
**BP_Gp7**	0.24	0.24	0.24	0.23	0.22	0.23	**n/c**

**Table 4.** Distances within (in bold) and between *Baetis maurus* (BM_Gp1-BM_Gp3) and Baetis cf. alpinus haplogroups (BM_Gp4-BM_Gp7). Haplogroup with Algerian sequences is underlined.

	**BM_Gp1**	**BM_Gp2**	**BM_Gp3**	**BM_Gp4**	**BM_Gp5**	**BM_Gp6**	**BM_Gp7**
**BM_Gp1**	**0**						
**BM_Gp2**	0.16	**0.003**					
**BM_Gp3**	0.15	0.16	**0.007**				
**BM_Gp4**	0.24	0.24	0.23	**0.043**			
**BM_Gp5**	0.26	0.24	0.21	0.23	**0.006**		
**BM_Gp6**	0.24	0.23	0.22	0.25	0.19	**0.06**	
**BM_Gp7**	0.25	0.26	0.22	0.25	0.19	0.19	**0.04**

**Table 5.** Distances within (in bold) and between *Acentrella* haplogroups. AC-Gp1: *Acentrella nadineae*. AC-Gp2: *Acentrella parvula*. AC-Gp3: *Acentrella turbida*. AC-Gp4: *Acentrella lapponica*. AC-Gp5: Acentrella cf. sinaica. AC-Gp6: *Acentrella sinaica*. Haplogroup with Algerian sequence is underlined.

	**AC_Gp1**	**AC_Gp2**	**AC_Gp3**	**AC_Gp4**	**AC_Gp5**	**AC_Gp6**
**AC_Gp1**	**0**					
**AC_Gp2**	0.19	**0.011**				
**AC_Gp3**	0.23	0.21	**0**			
**AC_Gp4**	0.22	0.22	0.24	**0.002**		
**AC_Gp5**	0.25	0.25	0.26	0.22	**n/c**	
**AC_Gp6**	0.26	0.25	0.24	0.25	0.19	**0.003**

**Table 6.** Distances within (in bold) and between *Cloeon* haplogroups. CO_Gp1-CO-Gp6: *Cloeon dipterum* sl. CO_Gp2: *Cloeon peregrinator.* Haplogroup with Algerian sequences is underlined.

	**CO_Gp1**	**CO_Gp2**	**CO_Gp3**	**CO_Gp4**	**CO_Gp5**	**CO_Gp6**	**CO_Gp7**	**CO_Gp8**	**CO_Gp9**	**CO_Gp10**	**CO_Gp11**
**CO_Gp_1**	**0.002**										
**CO_Gp_2**	0.09	**0.002**									
**CO_Gp_3**	0.10	0.10	**0.002**								
**CO_Gp_4**	0.11	0.11	0.07	**0.012**							
**CO_Gp_5**	0.11	0.11	0.08	0.08	**0.021**						
**CO_Gp_6**	0.09	0.09	0.1	0.1	0.1	**0.002**					
**CO_Gp_7**	0.15	0.15	0.17	0.17	0.16	0.16	**0.01**				
**CO_Gp_8**	0.18	0.19	0.19	0.19	0.20	0.20	0.15	**0.00**			
**CO_Gp_9**	0.19	0.21	0.2	0.19	0.19	0.20	0.20	0.22	**0.013**		
**CO_Gp_10**	0.17	0.19	0.17	0.18	0.18	0.19	0.19	0.20	0.11	**0.005**	
**CO_Gp_11**	0.17	0.19	0.16	0.17	0.17	0.17	0.18	0.18	0.18	0.19	**n/c**

**Table 7.** Distances within (in bold) and between *Procloeon* haplogroups (PC_Gp1-PC_Gp5) and *Centroptilum luteolum* group (PC_Gp6). PC-Gp1: *Procloeon stagnicola*. PC-Gp2 – PC-Gp3: *Procloeon bifidum*. PC-Gp4: *Procloeon pennulatum*. Haplogroup with Algerian sequences is underlined.

	**PC_Gp1**	**PC_Gp2**	**PC_Gp3**	**PC_Gp4**	**PC_Gp5**	**PC_Gp6**
**PC_Gp1**	**0.006**					
**PC_Gp2**	0.16	**0.027**				
**PC_Gp3**	0.18	0.19	**n/c**			
**PC_Gp4**	0.19	0.19	0.20	**0.016**		
**PC_Gp5**	0.19	0.20	0.21	0.20	**0.037**	
**PC_Gp6**	0.22	0.23	0.24	0.24	0.23	**0**

**Table 8.** Distribution of the Baetidae of the Tafna catchment. Distribution prior to study, based on ^1^Bauernfeind and Soldán (2012); ^2^Fauna Europa (Fauna Europaea 2020); ^3^Macadam et al. (2018).

**Operational Taxonomic Units**	**Implications from this study**
Acentrella cf. sinaica	New unnamed species in North Africa
*Baetis maurus*	Endemic to North Africa^1,2^; new unnamed species in Iberian Peninsula
Baetis cf. pavidus	North Africa and South of France^1,2^
Baetis (Rhodobaetis) atlanticus	First report for North Africa; known from Macaronesia and Atlantic Europe^1,3^
Baetis (Rhodobaetis) sinespinosus	Confirmation of North African endemism^1^
*Cloeon peregrinator*	First report for North Africa; known from Macaronesia^1^
*Procloeon stagnicola*	Confirmation of North African endemism^1^
