# Supplementary figures and images for: Reports of Baetidae (Ephemeroptera) species from Tafna Basin, Algeria and biogeographic affinities revealed by DNA barcoding
Source: Biodivers Data J. 2020 Aug 14;8:e55596. doi: 10.3897/BDJ.8.e55596 (PMC7442755; doi:10.3897/BDJ.8.e55596)

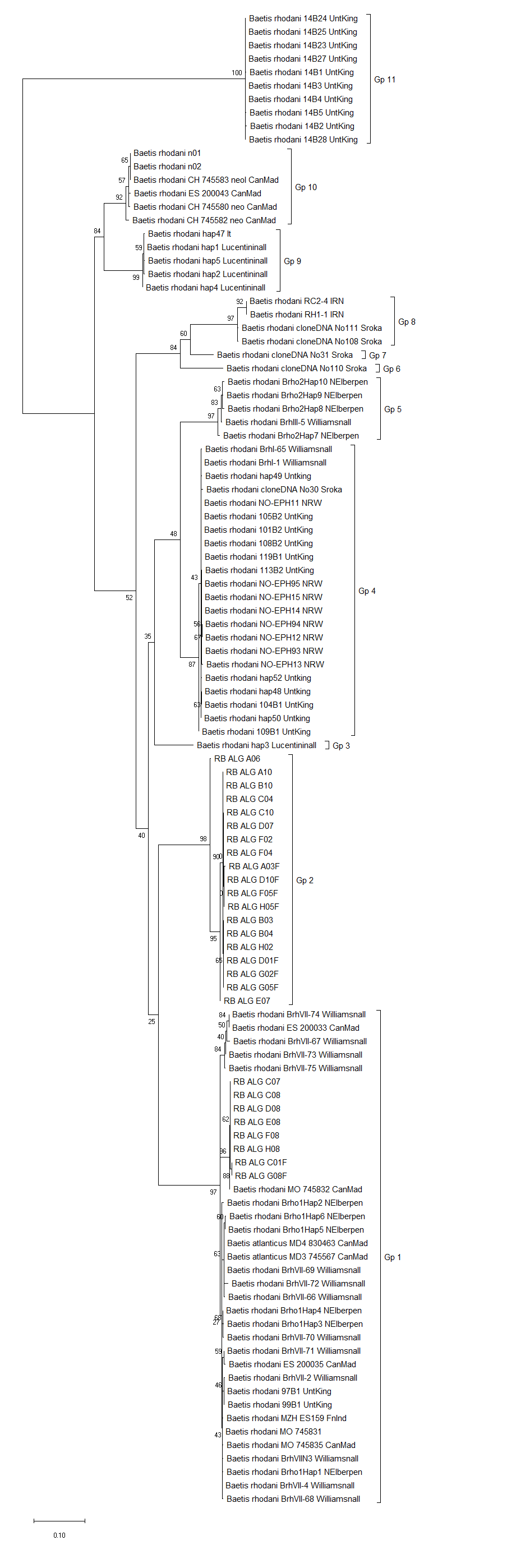

Supplement: Supplementary material 1 — Complete Maximum Likelihood tree including representative of Rhodobaetis using TN93 (+G+I) model [file bdj-08-e55596-s001.tif]

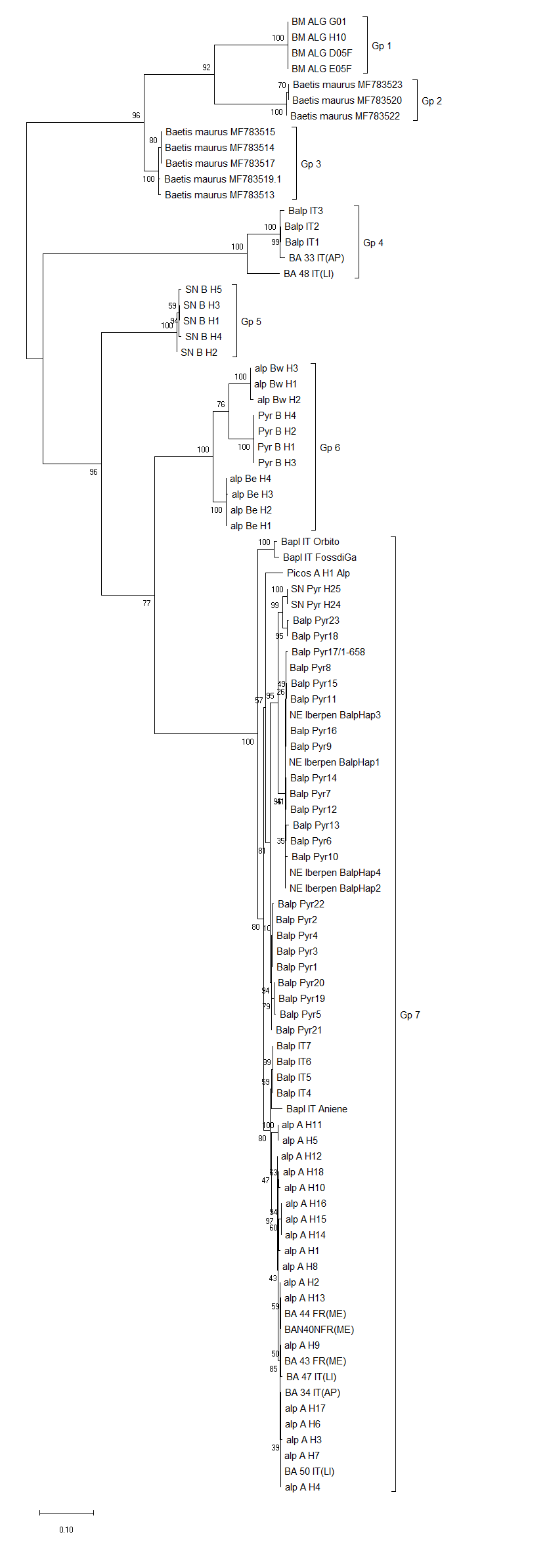

Supplement: Supplementary material 2 — Complete Maximum Likelihood tree including representative of Baetis maurus using TN93 (+G+I) model [file bdj-08-e55596-s002.tif]

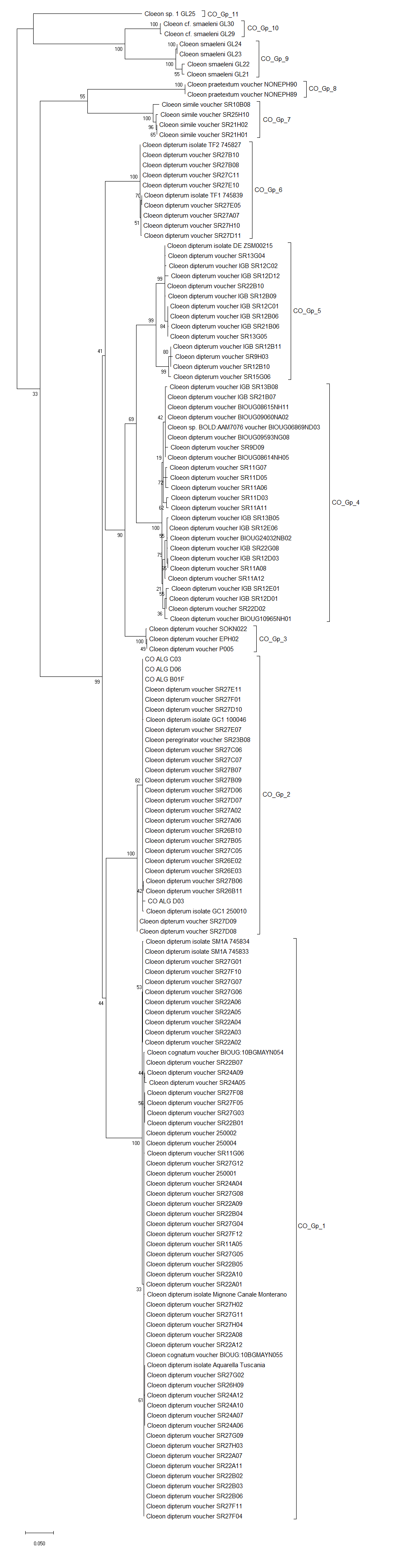

Supplement: Supplementary material 3 — Complete Maximum Likelihood tree including representative of Cloeon spp. using General Time Reversible model (+G+I) [file bdj-08-e55596-s003.tif]
